# Should annual cost of the drug inform reimbursement decisions? A perspective from China’s healthcare security system

**DOI:** 10.3389/fpubh.2025.1552798

**Published:** 2025-04-04

**Authors:** Xiaochen Peng, Haiyin Wang, Hui Sun, Xiaoxiao Qin, Zhe Huang

**Affiliations:** ^1^School of Business Administration, Shenyang Pharmaceutical University, Shenyang, Liaoning, China; ^2^Shanghai Health Development Research Center (Shanghai Medical Information Center), Shanghai, China

**Keywords:** basic medical insurance, binary logistic regression, annual cost, threshold, drug reimbursement, reimbursement decision-making, China, out-of-pocket

## Abstract

**Background:**

An increasing number of countries worldwide, including China, have adopted Health Technology Assessment (HTA) and pharmacoeconomic (PE) principles, either comprehensively or partially, to inform drug reimbursement decisions. While China has integrated the annual cost of the drug (ACD) as a key economic factor considered in decision-making, implicitly establishing a price ceiling for medical insurance coverage. However, the current approach lacks a robust theoretical foundation and quantitative evidence.

**Objective:**

This study aims to explore the rationale for incorporating ACD as a constraint in reimbursement decision-making framework, and to estimate a practical ACD threshold for China’s basic medical insurance (BMI) system.

**Methods:**

Binary logistic regression was employed to analyze the impact of ACD on patients’ financial burden. The outcome variable was the occurrence of financial barriers, with ACD serving as the primary independent variable. Covariates are factors of reimbursement benefits, including reimbursement caps and reimbursement rates. Average marginal effect analysis was performed to quantify the relationship between ACD and the likelihood of encountering barriers, suggesting a ACD threshold with practical implications for reimbursement decisions. Multicollinearity among variables was assessed using the Variance Inflation Factor (VIF). The model’s goodness of fit was assessed using the likelihood-ratio test and the Hosmer-Lemeshow test. Additionally, model performance was evaluated using the Receiver Operating Characteristic (ROC) curve.

**Findings:**

In China, patients face significant challenges in affording high-priced medications under BMI system. Failure to consider the payment capacity of the general population in drug reimbursement decision-making can result in an inequitable allocation of basic medical insurance funds. Logistic regression analysis revealed that for each 10,000 CNY (approximately 1,431 USD) increase in the ACD, the odds of outcome occurring increased by a factor of 1.1681 (95% CI: 1.1365–1.2006, *p* < 0.001). The highest average marginal effect was observed at a ACD value of 400,000 CNY (0.0228; 95% CI: 0.0199–0.0256, *p* < 0.0001). Furthermore, when ACD exceeded 440,000–450,000 CNY, the predicted probability of financial barriers surpassed 50% (*p* < 0.001).

**Conclusion:**

Incorporating the evaluation of ACD into the appraisal process is crucial for informing reimbursement decisions, especially in health insurance systems without robust safety net mechanisms for patients. This study innovatively estimated the value of ACD threshold, addressing the research gap and providing a methodological reference for quantitative research. Despite adopting maximized reimbursement benefits of BMI, the estimated ACD threshold appears unable to support innovative medications priced comparably to those in global markets. In the future, China should establish a risk-sharing mechanism and improve the level of medical reimbursement benefits to mitigate financial barriers for patients’ access to high-value medications.

## 1 Background

The rapid progress of medical technology and the booming emergence of innovative pharmaceuticals have presented new therapeutic opportunities for disease treatment. Nonetheless, the substantial costs associated with drug Research and Development (R&D) and patent protections often drive up prices. Meanwhile, limited healthcare resources have highlighted the growing challenges in resource allocation.

Health Technology Assessment (HTA) and pharmacoeconomics (PE) evaluation, as effective tools for evaluating ‘good value for money,’ have been increasingly adopted by numerous healthcare systems in informing drug reimbursement decisions. The elements evaluated encompass a variety of aspects, generally including clinical effectiveness, health economic evaluation (cost-effectiveness analysis, cost-utility analysis), social and ethical impacts, organizational impact and patient perspectives. Among diverse healthcare systems, the practical processes and evaluation methods in use demonstrate significant variation (1). Essentially, assessments regarding economic aspect include cost-effectiveness analysis/cost-utility analysis (CEA/CUA), budget impact analysis (BIA), reference prices of alternatives, and international reference pricing (2, 3).

**Figure 1:**
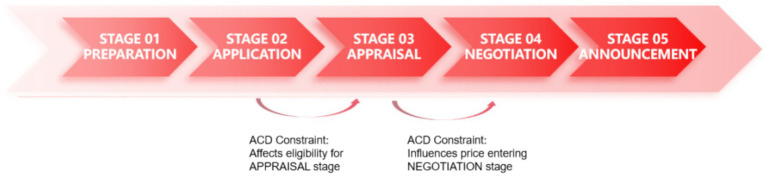
NRDL annual adjustment workflow and the role of ACD constraint.

In China, the annual adjustments to the National Reimbursement Drug List (NRDL) serve as the primary pathway for reimbursement of marketed drugs. This mechanism formally commenced in 2018 and incorporates HTA methodology and PE evaluations within a structured five-stage decision-making procedure (4, 5) (see Figure 1). The economic evaluations primarily take place in the third Stage. However, a distinctive feature of China’s approach, compared to other countries that have adopted HTA framework in decision-making, is the inclusion of the annual treatment cost of medicine as specified in the drug’s instructions as one of the economic value criteria (6–8), hereafter ACD (Annual Cost of the Drug) was used as the abbreviation to specifically refer to the annual cost that calculated according to the drug label. The ACD must not exceed a certain threshold to be eligible to enter the appraisal and negotiation stages. Based on the NRDL data from 2018 to 2023, the ACD for new entries has not surpassed 300,000 CNY. Consequently, this figure is regarded as an implicit ACD threshold, despite the absence of an official declaration or academic documentation.

However, the rationale for employing an annual cost threshold in decision-making, as well as the magnitude of this threshold, remains implicit and ambiguous. Our literature review reveals that the use of ACD constraints—particularly as threshold-based rules—is exceptionally rare in global reimbursement practices. Typically, cost-control measures in specific reimbursement decisions adopt a payer perspective, emphasizing resource allocation for defined disease groups through mechanisms such as budget impact analysis and restrictions on reimbursable treatment courses to improve cost predictability (9–11). A significant gap exists in academic research regarding the estimation of annual cost thresholds or constraints. Concurrently, there is an absence of robust evaluation standards incorporating the ACD perspective within the HTA framework.

To investigate whether the annual cost perspective should be systematically integrated into reimbursement decision-making—particularly within the HTA framework—we first conducted a review of the criteria for annual cost assessments in decision-making processes in the countries^1^ with well-established HTA agencies. We only found that in Netherlands, an annual cost of €50,000 or more per patient was one of the criteria used to identify a drug as alertly expensive to be “locked” (12). However, this “lock” mechanism is designed to initiate additional evaluation rather than serving as a criterion for denying reimbursement approval or implying a ceiling for price.

We further explored variations across healthcare systems to elucidate why decision-makers may overlook ACD considerations, selecting reference regions based on their insurance models: the hybrid system in the United States, the National Health Service (NHS) in the United Kingdom, and the social health insurance systems in Germany and Japan. Analysis of their financing models and benefit structures revealed two primary distinctions: (1) Lack of ceiling of co-payment or Out-of-Pocket (OOP) cap mechanism (13–18) (see Table 1). In China’s basic healthcare insurance system, there is no upper limit on OOP expenses for patients, while a maximum cap is set on the reimbursement payments. Therefore, patients may face unconstrained OOP costs, and an increase in the ACD will directly exacerbate the financial burden on patients, while insurers remain constrained by predetermined reimbursement ceilings. (2) Limited access pathways and risk-sharing. Compared to reference regions, China lacks flexible access pathways and risk-sharing schemes for patient access to innovative drugs. For instance, the U.K uses various strategies including commercial arrangements, managed access agreements and supplementary funding (e.g., the Cancer Drug Fund) to optimize access to innovative drugs (19), while Germany enables rapid patient access to innovative therapies with subsequent price adjustments made based on real-world data and economic evaluations (20). In contrast, China has not yet established a comprehensive post-market access management system, which includes mechanisms such as special catalog inclusion or price reassessment based on clinical outcomes. Furthermore, it lacks compliant pathways to implement rebates, hindering the adoption of outcome-based payment models.

**Table 1:** Rules regarding OOP expense cap for patients in reference regions.

Region	Health insurance system	Insurance benefit—rules regarding OOP cap	GDP per capita in 2022 (US dollars)^a^	OOP cap in GDP per capita^b^
The U.S.	Hybrid Model	Medicare: In terms of prescription drug coverage under Part D, the annual out-of-pocket maximum is capped at $8,000, with a specific limit of $2,000 that will take effect in 2025. Medigap (supplemental insurance) policies have an annual out-of-pocket limit that does not exceed $7,060 for individuals and $3,530 for couples.	77,247	3% of GDP per capita
England	National Health Service System	With the exception of a few specific items, the majority of healthcare services provided by the NHS are available for free.	45,564	NA
German	Social Health Insurance System	The annual out-of-pocket maximum is set at 2% of the assessed income for regular beneficiaries and 1% for those with chronic illnesses. Combined with a reduction mechanism: the first family member living with the insured individual can deduct 15% of the household income, with an additional 10% deduction for each subsequent member. For outpatient visits and medication purchases, insured individuals pay a minimum of €5 and a maximum of €10 per transaction. For inpatient care, a daily fee of €10 is charged, with an annual out-of-pocket maximum of €280.	48,718	2% of GDP per capita^c^
Japan	Social Health Insurance System	A tiered limit system is implemented based on population age and income. The specific limits are calculated using a formula. For individuals with a monthly salary of less than 260,000 yen, as well as for any individuals who have incurred high medical expenses for three months or more in the year preceding the month of treatment, a fixed limit will apply starting from the fourth month. The fixed limit mentioned above ranges from 24,600 yen to 140,100 yen.	34,017	3% of GDP per capita

NA, Not Applicable.

a All GDP data in the study are sourced from the World Bank database.

b Exchange rate data is sourced from the International Monetary Fund (IMF), the highest OOP cap was used for the calculations.

c The average gross annual salary in Germany in 2022 was €49,260.

We additionally investigated the practices in South Korea, an Asia-Pacific nation that also has implemented HTA, where pharmaceutical reimbursement prices are generally lower compared to other developed nations. In South Korea, a significant portion of South Korea’s population—approximately 70%—is covered by private insurance, which complements public insurance by reimbursing co-payments and covering services not included in the public scheme. Risk-sharing agreements (RSA) have been employed to enhance patient access to innovative treatments. While no ACD constraints were found in reimbursement decision-making process (21).

**Figure 2:**
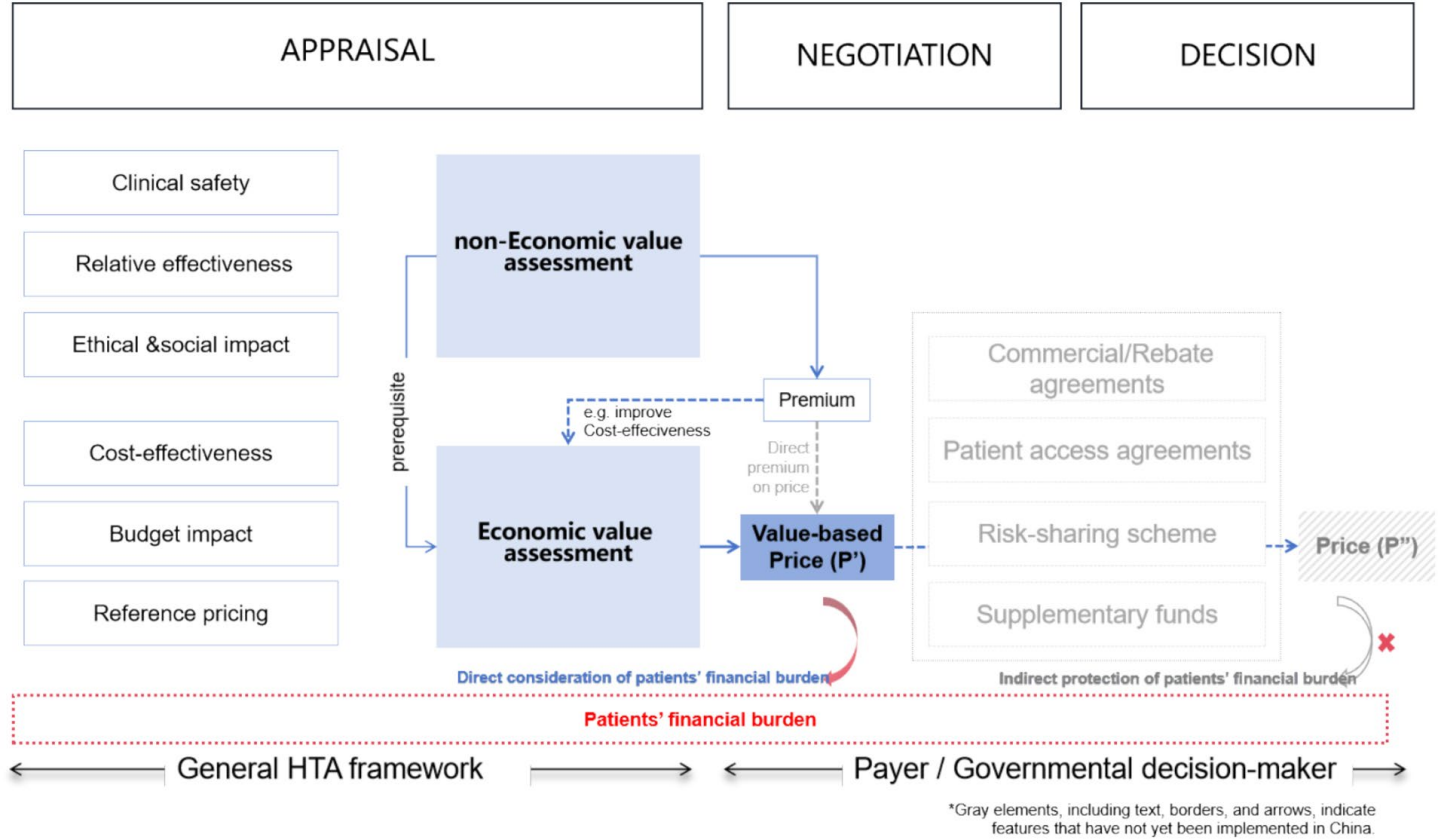
Road map of HTA/PE informed decisions.

China extends additional financial protection to impoverished households in accessing healthcare, there remains an absence of regulatory provisions to ensure financial security for the broader population and to avert poverty stemming from medical expenses. However, the lack of an OOP expenditure cap and the absence of well-established, comprehensive access pathways for innovative pharmaceuticals mean that the financial burden on patients must be sensitive to drug prices. China have integrated the multi-dimensional value of medicines into their drug reimbursement decision-making processes to enhance value-based pricing (see Figure 2). Despite the HTA framework’s consideration of the patient perspective, which includes factors such as quality of life, financial burden, and caregivers’ burden, it lacks quantitative and applicable criteria for managing the cost burden of pharmaceuticals. This is primarily due to the fact that in the majority of countries employing HTA, the predominant economic burden is derived from indirect expenses like days lost at work, as opposed to the cost-sharing for medines (22–24). At the same time, there is a lack of flexible pathways to indirectly protect patients from financial difficulties. Therefore, the appraisal process for drug reimbursement in these contexts requires a more pragmatic approach compared to those applied in high-welfare regions. In addition to evaluating the incremental benefits of new drugs compared to existing treatments (CUA/CEA), the affordability of the funds (BIA), and considering reference pricing, the payer—who is both the decision-maker and major purchaser with bargaining power—must also manage annual treatment costs from the patient’s perspective. Therefore, ACD constraints particularly hold relevance in decision-making process within the context of China’s healthcare insurance system.

Clearly, while ACD is directly related to the price of drug, it does not exhibit a direct correlation with the existing evaluation method such as CUA or BIA. A drug having an unacceptable high ACD amount does not necessarily mean that it will yield a negative result in CUA or BIA. CUA is an incremental analytical approach that assesses both costs and outcomes associated with a given intervention relative to its alternatives. The time horizon in CUA ought to exceed the treatment duration of the appraised intervention, and the costs extend beyond the direct cost of the drug. While BIA are contingent upon various parameters, including the size of patient population, alternative drug prices, the treatment duration, market share substitution, and anticipated changes in the utilization of medical resources. In short, establishing a direct correlation between the ACD and CUA or BIA within the current framework presents significant methodological challenges.

Given that the ACD constraint, functioning as a threshold, independently affect the final pricing of pharmaceuticals within the decision-making process, this study aimed to investigate the maximum “acceptable” ACD as the threshold for drug reimbursement decisions.

## 2 China’s basic medical insurance system and reimbursement benefits

China has successfully operated a universal basic medical insurance (BMI) system achieving a coverage rate consistently exceeding 95% of its population (25). In China’s healthcare system, basic medical insurance holds a dominant position and serves as the primary means for reimbursement of medical costs. Commercial health insurance typically acts as a supplementary form of coverage, with a small payout ratio, accounting for approximately 5% of direct medical expenditures (26). The BMI system in China comprises two main components: the Urban Employee Basic Medical Insurance (UEBMI) and the Urban and Rural Resident Basic Medical Insurance (URRBMI). The UEBMI provides essential healthcare coverage to urban employees and retirees, while the URRBMI aims to address the healthcare needs of urban and rural residents, including students. The former is complemented by the Medical Subsidy for Large Medical Expenses (MSLME), while the latter is supplemented by the Major Illness Insurance (MII). MSLME is a crucial component of UEBMI, providing supplementary reimbursement for significant medical expenses within the UEBMI framework. Importantly, it does not function as a separate insurance coverage that necessitates distinct participation or additional premium payments. Similarly, the relationship between MII and URRBMI reflects this same structure. These programs are designed to provide extensive coverage for insured individuals, with the goal of mitigating the financial burden associated with substantial medical expenses. In principle, the ceiling for annual reimbursement payments under these combined schemes should reach approximately six times the local average annual wage for employees (UEBMI with MSLME) and six times the annual per capita disposable income for residents (URRBMI with MII), respectively (27).

Specific reimbursement policies exhibit variation across regions, influenced by disparities in economic development levels and the financial capacity of local insurance funding (28). The reimbursement rules and insurance benefits associated with UEBMI (with MSLME) and URRBMI (with MII) are predominantly established at the municipal level, although some are determined at the county level. Consequently, there are hundreds of rules effective in the BMI system, with premium standards, reimbursement rules, and insurance benefits standardized within each city and municipality. In general, the UEBMI offers more generous benefits compared to the URRBMI, such as higher reimbursement rates and a larger annual reimbursement payment maximum.

In China, drug reimbursement is facilitated through a catalog-based system, with the unified NRDL acting as the comprehensive national catalog that delineates which medications are eligible for insurance coverage. Within the context of China’s medical insurance system, the OOP expense for patients using drugs from NRDL are influenced by several factors, including,

1. Deductible amount: The initial amount patients must pay before insurance coverage begins.
2. Reimbursement rate: The percentage of the medical cost that is reimbursed by the insurance.
3. Annual reimbursement cap: The maximum amount that can be reimbursed in a year.
4. Drug co-payment ratio: This refers to the percentage of the drug cost that patients are required to pay upfront.

For example, if the treatment cost of a drug from NRDL is below the annual reimbursement cap, the amount payed by basic medical insurance (BMI), can be calculated using the following formula:


$$\begin{array}{l} \mathrm{Reimbursement payment}\\ =\left(\mathrm{drugs}\;{\mathrm{cost}}^{*}\left(1-co-\mathrm{payment ratio}\right)-\mathrm{deductible}\right)\\ {}*\mathrm{reimbursement rate}\text{.} \end{array}$$

Category A drugs are subject to a zero co-payment ratio, whereas Category B drugs have a co-payment ratio that typically falls between 10 and 30%. This implies that for Category B drugs, patients must first cover a certain percentage of the drug cost before the remaining cost enters into a cost-sharing phase.

## 3 Methodology

### 3.1 Model structure

This study aimed to investigate the effect of changes in ACD on patients’ financial stress under current benefit level, specifically by determining if a given ACD expense will have a catastrophic impact on the general household’s finances and affordability. Logistic regression, primarily used for risk prediction, allows for hypothesis testing to assess the significance and relative importance of variables. Notably, the binary logistic regression model effectively captures the impact of continuous variables on event occurrence; in binary logistic regression, average marginal effects (AME) analysis refer to the average change in the probability of the outcome variable associated with a one-unit change in a predictor variable; thus, identifying the value of ACD that maximizes the probability change could provide a reference for threshold setting. We employed a dichotomous variable—the occurrence of patient financial barriers—evaluated based on whether catastrophic household expenditures occurred as the outcome variable. The function in our study was structured as follows:


$$\begin{array}{l} \log\left(P\left(\mathrm{cata}=1\right)/\left(1-P\left(\mathrm{cata}=1\right)\right)\right)=\beta 1\cdot X_{ACD}+\beta 2\cdot X_{\mathrm{UEBMIcap}}\\ +\beta 3\cdot X_{\mathrm{MSLMEcap}}+\beta 4\cdot X_{\mathrm{UEBMIrate}}+\beta 5\cdot X_{\mathrm{MSLMErate}}\\ +\beta 6\cdot X_{\mathrm{Drugcopay}}+\mathrm{Constant}\text{.} \end{array}$$

Where X_ACD_ represents the key independent variable ACD. X_UEBMIcap_, X_MSLMEcap_, X_UEBMIrate_, X_MSLMErate_, X_Drugcopay_ represents the independent variable regarding reimbursement benefit, including reimbursement cap of UEBMI, reimbursement cap of MSLME, reimbursement rate of UEBMI, reimbursement rate of MSLME, Drug co-payment ratio. *β* measures the impact of the change of each independent variable on outcome variable.

The odds ratio of risk associated with each unit increase in ACD and the occurrence of financial barriers was examined. We determined ACD thresholds as the value maximizing the marginal effect of ACD and the value above which over 50% of observations faced financial barriers. The analysis was conducted using Stata15.

### 3.2 Variable selection

#### 3.2.1 Outcome variable

Catastrophic health expenditure refers to healthcare costs that are so high they threaten a household’s financial stability, potentially leading to severe financial consequences. The World Health Organization (WHO) recommends, and academia widely adopts, a threshold of 40% to define catastrophic health expenditure (29). In this study, the occurrence of catastrophic household expenditure was specifically defined as OOP expenses exceeding 40% of a household’s annual disposable income. Data on per capita disposable income and average household size for each province and municipality are sourced from the “China Statistical Yearbook 2023” (30).

#### 3.2.2 Independent variable

The ACD variable is a key explanatory variable and the range should represent the maximum value it can attain, particularly in the context of an HTA framework where no annual cost constraints are imposed on the appraisal of innovative drugs. In China, the ACD values of drugs listed in NRDLare generally below 300,000CNY (42,857USD^2^), resulting in a lack of localized, reasonable reference values to support the model-based evaluation of ACD thresholds. Therefore, an investigation was conducted to identify the achievable prices and ACD amounts for innovative drugs on a global scale. We selected several high-priced innovative drugs that have been launched in China but failed to be included in the NRDL (discontinued prior to the appraisal stage) in recent years and examined their reimbursement status and prices across four reference regions. Considering that medical insurance settlements are conducted on an annual basis, this study only takes into account the treatment costs for the current year and does not consider the treatment duration or discount rate.

A number of innovative pharmaceuticals were reviewed, including CAR-T therapies, anti-cancer drugs, and orphan drugs. Ultimately, the following drugs were selected for case study: Axicabtagene (Car-T), fam-trastuzumab deruxtecan-nxki, Inotuzumab ozogamicin, Emicizumab, and Selumetinib (Selumetinib were successfully included in the 2024 NRDL through price negotiation, with the ACD dropping to less than 300,000CNY (42,857USD) after the inclusion) (31–34).

The publicly available prices of the five drugs in the reference regions were collected and their ACD amounts were calculated based on their recommended usage as specified in the prescribing information. These amounts were then converted into multiples of the respective GDP per capita for each region. The highest ACD amount was nearly 12 times the GDP per capita (see Table 2). As a reference, 12 times China’s GDP per capita, approximately 1,200,000 CNY, was used as the upper bound of the ACD range. Consequently, the ACD variable ranged from a minimum of 0 CNY to a maximum of 1,200,000 CNY (171,428USD), with increments of 50,000 CNY (7,143USD) between each value.^3^

**Table 2:** ACD of case study drugs in terms of multiples of respective GDP per capita.

Brand name	Generic name	The U.S.	Germany	England	Japan
YESCARTA	Axicabtagene	5.9	8.5	NA	6.8
ENHERTU	Fam-trastuzumab deruxtecan-nxki	2.6	3.4	2.5	2.0
BESPONSA	Inotuzumab ozogamicin	3.5	7.9	3.8	4.3
HEMLIBRA	Emicizumab	7.5	11.4	NA	9.9
KOSELUGO	Selumetinib	NA	4.7	5.2	9.2

“NA” indicates that reimbursement information for this drug in the specified region was not available, with data collection concluding in June 2023. GDP per capita for 2022 in US dollars: United States: 77,247USD, Germany: 48,718USD, United Kingdom: 45,564USD, Japan: 34,017USD, China: 12,662USD.

The covariates were selected based on factors related to reimbursement benefits (as introduced in Section 2), as they were determinants of both the reimbursement payments and OOP expenses, including deductibles (UEBMI and UEBMI, respectively), drug co-payment ratio, reimbursement rates and caps (UEBMI and UEBMI, respectively). Given that the benefits were coordinated at the municipal level (35), we collected the benefit rules for the UEBMI and its supplementary MSLME from 31 provincial capital cities (including 4 municipalities). These cities typically offer more generous cost-sharing provisions compared to other cities within the same province, making them representative of the highest level of BMI benefits in China. In addition, as reimbursement rates varied according to the level of the hospital, we utilized the rates applicable to tertiary hospitals, as high-cost medications were often prescribed for complex conditions such as cancer and rare diseases.

Therefore, we created 31 study objects representing the 31 provinces in China, each characterized by its household payment capacity and corresponding reimbursement benefits (Supplementary Table 1-1).

### 3.3 Data processing

#### 3.3.1 OOP calculation

For each study object, we calculated the out-of-pocket (OOP) expenses when confronted with gradually increasing amounts of ACD. As a result, with 31 reimbursement rule sets and 23 ACD values, the sample consisted of 713 observations (31 reimbursement rules × 23 ACD values = 713 observations, as elucidated in Supplementary Tables 1-2, 1-3).

The General Steps for OOP Calculation were as follows^4^:

Step1: for costs below the UEBMI reimbursement cap:
Reimbursement payment 1 = (ACD’ × (1−co-payment ratio for Category B drugs)-deductible) × UEBMI reimbursement rate.
Step2: for costs above the UEBMI reimbursement cap, below the MSLME reimbursement cap.
Reimbursement payment 2 = ((ACD”−MSLME reimbursement cap)−MSLME deductible) × MSLME reimbursement rate.
Step3: Then, OOP = ACD−Reimbursement payment 1−Reimbursement payment 2.

In some cases, a tiered reimbursement approach was implemented in the MSLME program, where increasing reimbursement rates were applied to each tier. This structure continues until the total treatment costs eligible for reimbursement reach a predetermined cap. This tiered approach ensures that patients receive higher reimbursement rates for larger treatment costs, up to the specified maximum limit. The reimbursement payment was calculated according to the rules, which involve summing the costs in each tier and then multiplying by their corresponding reimbursement rates.

The reimbursement payment calculation for expenses that enter the MSLME phase was performed according to the specific reimbursement rule established in each object.

#### 3.3.2 Outcome variable processing

OOP costs were considered catastrophic when they surpassed 40% of the household’s disposable income. Household disposable income was calculated based on the per capita disposable income of each province and municipality, using an average household size of 2.62 persons (36). The outcome variable (denoted as “cata”) that represented the occurrence of catastrophic expenditure was thus calculated using a threshold where the OOP expenses accounted for 40% of the household’s disposable income, specifically:


$$\mathrm{When}\;\left(OOP/\mathrm{per}\;{\mathrm{capita disposable income}}^{*}\;2.62\right)>40\%,\mathrm{cata}=1.$$

$$\mathrm{When}\;\left(OOP/\mathrm{per}\;{\mathrm{capita disposable income}}^{*}\;2.62\right)\leq 40\%,\mathrm{cata}=0.$$

#### 3.3.3 Classification of reimbursement rates

In this study, all variables related to reimbursement rates were transformed into categorical variables to mitigate coefficient bias and enhance model interpretability, with classifications designated as Levels 1, 2, and 3. For MSLME reimbursement rules, some of which feature tiered structures, both the highest reimbursement rate and the span between the highest and lowest tiers were considered in the categorization process.

The highest reimbursement rate for each MSLME rule was classified into three levels: A, B, and C. Similarly, the span between the highest and lowest tiers was also classified into three levels: A, B, and C. The grading was designed to ensure approximately equal sample sizes across levels, thereby preserving classification validity (see Table 3). The overall categorization of each MSLME rule was determined based on the following criteria:

1. If at least one item was classified as A, the rule was assigned to Level 3.
2. If at least one item was classified as C and none were classified as A, the rule was assigned to Level 1.
3. All other cases were classified as Level 2.

**Table 3:** The classification rules of MSLME reimbursement rate.

	Classification	Counts (*N* = 31)
The highest reimbursement rate		
≥95%	A	11
>80%, < 95%	B	13
≤80%	C	7
The difference between the highest and lowest reimbursement rate		
=0	A	19
>0, <15%	B	6
≥15%	C	6

According to the established criteria, a Level 3 categorization indicates a higher level of benefits, characterized by a higher reimbursement rate and fewer tiers. An analysis of 31 sample reimbursement rules for the MSLME reimbursement rate variable revealed a distribution in which 8 rules were classified as Level 3, 16 rules as Level 2, and 7 rules as Level 1.

In contrast, the UEBMI reimbursement rate is a fixed value for each rule. As a result, the same 31 sample reimbursement rules for the UEBMI reimbursement rate variable were directly categorized into three levels, with 9 rules assigned to Level 3, 18 rules assigned to Level 2, and 4 rules assigned to Level 1 (see Table 4).

**Table 4:** The classification rules of UEBMI reimbursement rate.

The reimbursement rate of UEBMI	Level	Counts (*N* = 31)
≥90%	3	9
≥85%, <90%	2	18
<85%	1	4

## 4 Results

### 4.1 Descriptive analysis

The statistical summary of UEBMI (MSLME) reimbursement rules for the year 2022 in each provincial capital cities and municipalities in China was presented in Table 5.

**Table 5:** Statistical summary of UEBMI (MSLME) reimbursement benefit in 31 cities.

Variable	*n*	Mean ± SD	Median	Skewness	Min	Max
UEBMI deductible	31	0.086 ± 0.028	0.08	0.7869	0.02	0.17
MSLME deductible	31	0.911 ± 0.001	0.7	0.6930	0	3.04
UEBMI reimbursement rate	31	0.867 ± 0.047	86%	−0.5330	75%	96%
UEBMI reimbursement cap	31	29.661 ± 21.678	24	0.7858	4.7	87
MSLME reimbursement cap	23	50.708 ± 35.415	43	3.4653	20	200
MSLME lowest reimbursement rate	31	0.835 ± 0.128	90%	−0.7479	60%	100%
MSLME highest reimbursement rate	31	0.884 ± 0.081	90%	−0.8630	70%	100%
Co-payment ratio for Category B	31	0.110 ± 0.044	10%	−0.7094	3%	20%

Across 31 cities, UEBMI inpatient reimbursement deductibles varied significantly, from as low as 200 CNY (28USD) to as high as 1,700 CNY (243USD). Reimbursement caps were equally diverse, ranging from 50,000 CNY (7,143USD) to a substantial 870,000 CNY (124,286USD). The reimbursement rate for inpatient care at tertiary hospitals ranged from 75 to 96%. In the realm of MSLME reimbursement rules, deductibles spanned from 0 CNY to 30,400 CNY (4,343USD). Caps ranged from 200,000 CNY (28,571USD) to a remarkable 2,000,000 CNY (285,710USD), with 8 cities offering no cap at all. Additionally, 12 cities implemented a tiered reimbursement mechanism, with the minimum tier starting at 10,000 CNY (1428USD). Overall reimbursement rates range from 60% to a full 100%. Regarding co-payment ratio for Category B, 25 out of the 31 cities featured ratios not exceeding 10%.

According to national economic statistical data in China for 2022, among the 31 provinces and municipalities in China, the highest per capita disposable income was 79,610 CNY, the lowest was 23,273 CNY, with an average of 36,592 CNY (median value of 30,957 CNY). Correspondingly, six times the average per capita disposable income was 219,498 CNY (median value of 185,740 CNY). Regarding the average wage of employed persons, the highest wage reached146,196 CNY, while the lowest was 71,580 CNY, with an average of 89,982 CNY. Six times the average wage was 539,893 CNY (see Table 6). The annual reimbursement payment maximum, namely, the sum of UEBMI and MSLME payment cap, for each of the 31 cities was examined. It was found that all but one, Inner Mongolia (Hohhot), matched or exceeded six times the local average wage of employed persons.

**Table 6:** Statistical summary of per capita disposable income and average wage of employed persons in China in 2022.

	Per capita disposable income CNY (USD)	Average wage of employed persons CNY (USD)	Six times the average wage CNY (USD)
Max	79,610 (11,373)	146,196 (20,885)	877,176 (125,311)
Min	23,273 (3,325)	71,580 (10,226)	429,480 (61,354)
Ave	36,583 (5,226)	89,982 (12,854)	539,893 (77,128)
Median	30,957 (4,422)	84,480 (12,068)	506,880 (72,411)

### 4.2 Binary logistic regression

The variable “MSLME cap” and “Co-payment ratio for Category B drugs” were not statistically significant in the model. They were excluded from the final analysis because the model’s fit slightly improved after removing these variables, as determined through AIC and BIC testing. A linear regression model was initially conducted (using OOP expenses as the dependent variable) to assess multicollinearity among the independent variables. This was evaluated using the Variance Inflation Factor (VIF) test. All independent variables had VIF values less than 3, which is generally considered acceptable, indicating low levels of multicollinearity.

The fitted logistic regression model demonstrated strong goodness-of-fit metrics. The likelihood-ratio chi-squared test yielded a value of 686.98 (*p* < 0.001), indicating the model significantly outperformed a null model without predictors. The pseudo R-squared value of 0.7262 indicates the model explained a substantial portion of the variability in the outcome variable. Further assessment of model fit was conducted using the Pearson chi-square test and the Hosmer-Lemeshow test. The Pearson chi-square test resulted in a *p*-value of 0.3701, while the Hosmer-Lemeshow test yielded a *p*-value of 1.0000. Both tests had *p*-values greater than 0.05, indicating no statistically significant discrepancy between the model-predicted probabilities and the actual observed outcomes. These results confirm that the logistic regression model provides a good fit to the data, thereby validating its use in analyzing the association between the independent variables and the binary outcome.

The results of the logistic regression analysis were presented in Table 7. The odds ratio (OR) for the variable ACD was 1.1681 (95% CI: 1.1365–1.2000), indicating that for each unit (10,000 CNY, approximately 1428USD) increase in ACD, the odds of the outcome occurring increased by a factor of 1.1681, and this effect was statistically significant (*p* < 0.001). Conversely, the OR for variable UEBMI cap was 0.9005 (95%CI: 0.8794–0.9221), suggesting that for each unit increase of 10,000 CNY (1428USD) in the UEBMI reimbursement cap, the odds of the outcome occurring are multiplied by 0.9005. Thus, higher levels of UEBMI reimbursement cap were associated with a significant decrease in the likelihood of outcome occurring.

**Table 7:** The odds ratio results of the logistic regression analysis.

Variable	Odds ratio	SD	*z*	*P*	[95% Conf. interval]
ACD	1.16812	0.0163438	11.11	0.000	[1.136522, 1.200596]
UEBMI cap	0.900515	0.0108929	−8.66	0.000	[0.8794164, 0.9221197]
UEBMI rate					
2	0.1417644	0.0753386	−3.68	0.000	[0.0500275, 0.4017222]
3	0.2461945	0.1385821	−2.49	0.013	[0.0816845, 0.7420225]
MSLME rate					
2	0.4786756	0.1995453	−1.77	0.077	[0.2114468, 1.083631]
3	0.0640387	0.0341475	−5.15	0.000	[0.0225193, 0.1821089]
Constant	0.2435322	0.1552002	−2.22	0.027	[0.0698386, 0.8492137]
LR chi^2^		686.98	*P*		0.0000
Log likelihood		−129.5266	Pseudo R^2^		0.7262

For categorical independent variables, the analysis revealed that the likelihood of the outcome occurring was significantly lower for Level 2 compared to Level 1 of the UEBMI rate, and for Level 3 compared to Level 2 of the MSLME rate. However, the effect of Level 3 compared to Level 2 of the MSLME rate was not statistically significant (*p* > 0.05), indicating that Levels 2 and 3 did not differ significantly in their impact on the outcome variable. Therefore, based on the classification rules for MSLME rate Levels 1–3, the findings indicated that when the highest MSLME reimbursement rate was larger than 90% and/or no tiered scheme was applied, the odds of the outcome occurring were significantly lower.

The average marginal effects (AME) was calculated to demonstrate the average change in the probability of the outcome associated with a one-unit change in each variable. The AME for ACD was 0.0086 (SE: 0.00003), indicating that, on average, a one-unit increase in ACD is associated with a 0.86 percentage point increase in the probability of the outcome. Similarly, a one-unit increase in the UEBMI reimbursement cap corresponded to a 0.58 percentage point decrease in the probability of the outcome (−0.0058, SE: 0.00042) (see Table 8).

**Table 8:** The results of average marginal effects analysis.

	dy/dx	Std. Err.	*z*	*P* > |*z*|	[95% Conf. interval]
ACD	0.008633	0.0000279	309.31	0.000	[0.0085783, 0.0086877]
UEBMI cap	−0.0058215	0.0004247	−13.71	0.000	[−0.0066539, −0.0049892]
UEBMI rate					
2	−0.108246	0.027697	−3.91	0.000	[−0.1625311, −0.0539609]
3	−0.0775191	0.0302765	−2.56	0.010	[−0.1368599, −0.0181783]
MSLME rate					
2	−0.0407987	0.0227301	−1.79	0.073	[−0.0853489, 0.0037515]
3	−0.152929	0.0263072	−5.81	0.000	[−0.2044901, −0.1013678]

**Figure 3:**
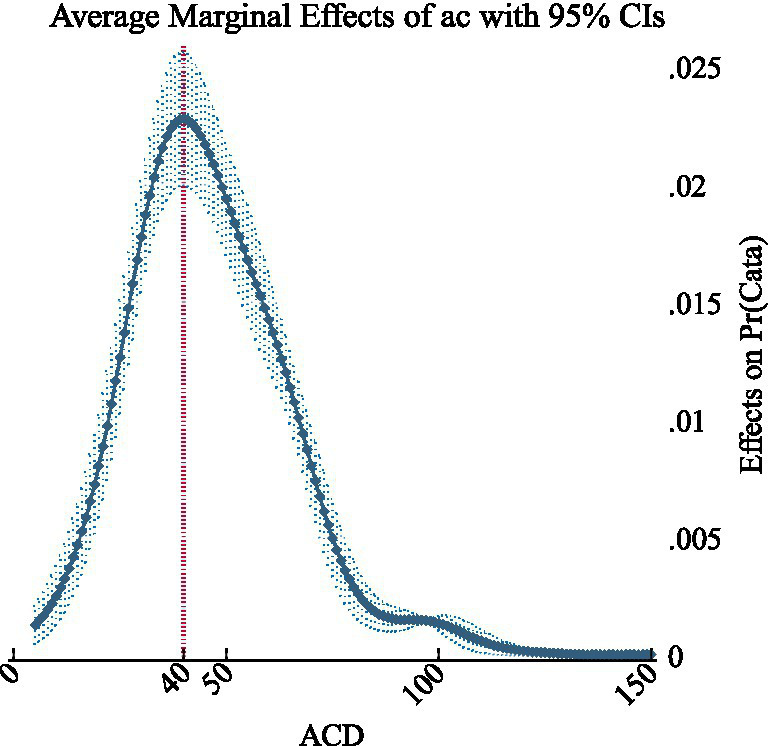
The curve of ACD average marginal effects.

For the ACD variable, the maximum marginal effect was observed at an ACD value of 40,000 CNY (5,714USD), with an marginal effect of 0.0228 (95% CI: 0.01994, 0.0256, *p* < 0.001). This marked the point where ACD has the greatest influence on the outcome. At this threshold, an increase from 39,000 CNY to 40,000 CNY in ACD were associated with a significant rise in the probability of the outcome variable being 1 (see Figure 3). This threshold represented the point at which changes in ACD have the most substantial impact on the probability of experiencing financial barriers. Thus, it serves as a robust reference for setting the ACD threshold in practical applications.

**Figure 4:**
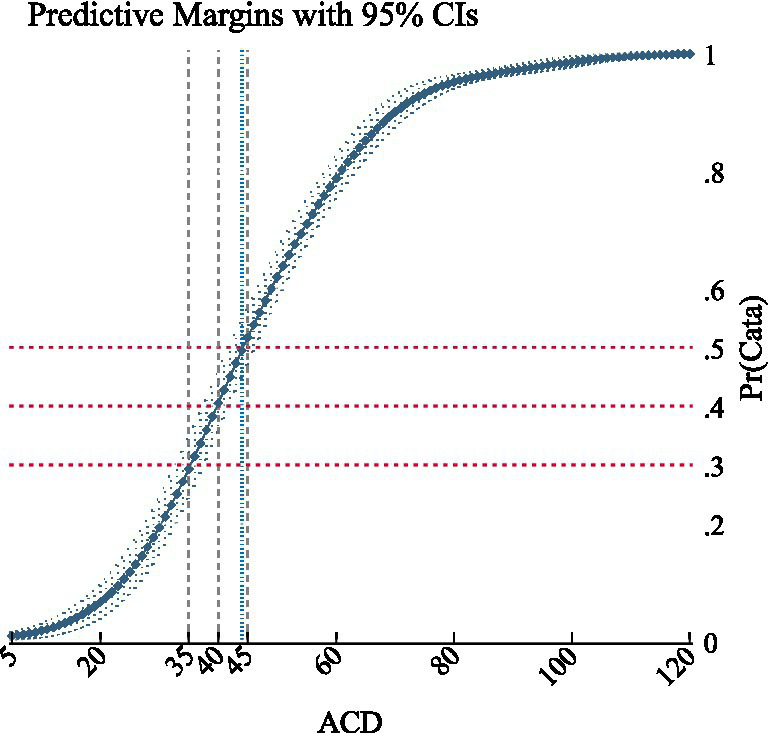
The curve of ACD cumulative effect.

Moreover, the analysis showed that the cumulative effect reached 50% at the range of 440,000 to 450,000 CNY (62,857 to 64,286USD) of ACD, corresponding to a predicted probability of 0.495 to 0.517 (95%CI:0.470–0.563, *p* < 0.001). This implies that ACD values higher than 450,000 CNY (64,286USD) are associated with a predicted probability greater than 50% of experiencing the outcome. Additionally, the cumulative effect reached 40% (95% CI: 0.355–0.454, *p* < 0.001) within the range of 390,000 to 400,000 CNY (55,714 to 57,143USD), and 30% (95% CI:0.264–0.365, *p* < 0.001) in the range of 350,000 to 360,000 CNY (50,000 to 51,428USD) (see Figure 4).

Since the key independent variable ACD was a continuous variable with values that fully cover the range of data fluctuation, we employed subgroup analysis to provide additional perspectives and informational references for the results. The subgroup analysis was performed on 12 sample cities in provinces where the per capita disposable income is below 30,000 CNY (4,286USD). Notably, the ACD value at which the maximum average marginal effect was observed decreased to 320,000 CNY, or 45,714USD (AME = 0.04166, *p* < 0.001). Furthermore, the ACD value corresponding to a cumulative effect of 50% fell below 350,000 CNY, or 50,000USD (95%CI: 0.4116–0.6000, *p* < 0.001). The detailed results were reported in Supplementary file 2.

**Figure 5:**
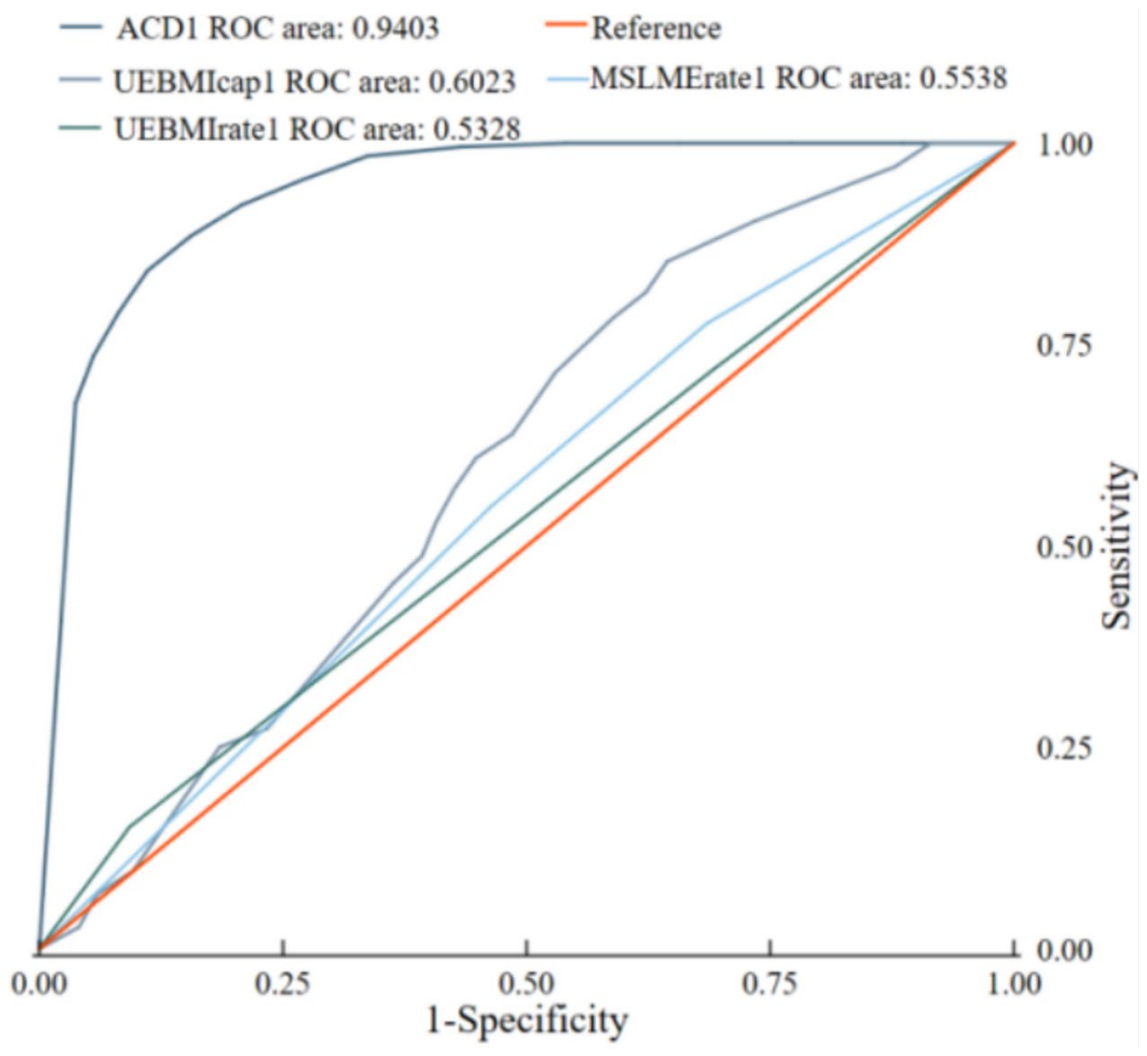
The ROC curve.

Receiver Operating Characteristic (ROC) analysis was conducted to evaluate the diagnostic performance of the logistic regression model. The Area Under the Curve (AUC) was computed to summarize the overall performance of the model. Among all variables, the variable ACD achieved the highest AUC value of 0.9403. Among all variables, ACD achieved the highest AUC value of 0.9403, indicating that ACD is the strongest predictor of the dependent variable and performs exceptionally well in predicting the outcome (see Table 9 and Figure 5).

**Table 9:** ROC curve analysis table for 4 independent variables.

Obs		AUC	Std. Err.	Asymptotic normal [95% Conf. interval]
ACD	713	0.9403	0.0094	[0.92186, 0.95882]
UEBMI cap	713	0.6023	0.0227	[0.55782, 0.64678]
MSLME rate	713	0.5538	0.0205	[0.51366, 0.59402]
UEBMI rate	713	0.5328	0.0193	[0.49487, 0.5707]
chi^2^(3) =486.53; Prob>chi^2^ = 0.0000				

## 5 Discussion

In recent years, China’s pharmaceutical market has emerged as the world’s second largest, driven by the growth of biopharmaceuticals, innovative drugs, and the increasing healthcare demands from an aging population. The annual growth rate of China’s pharmaceutical market has surpassed the global average. China’s BMI system, as the primary payer for pharmaceuticals, plays a critical role in determining drug inclusion and negotiated price in the NRDL through its adjustment rules and evaluation criteria. Similar to the reference regions, China utilizes HTA framework and pharmacoeconomic evaluation to inform drug reimbursement decisions. However, a crucial distinction is that China’s BMI system lacks OOP expense caps. Since 2020, the proportion of OOP expenditures in China’s total healthcare expenditures has steadily decreased to approximately 27% (37), nevertheless, this figure remains significantly higher compared to the reference regions. In 2023, the national BMI fund disbursed 2.8208 trillion CNY (0.40 trillion USD), while the OOP expenditures in China totaled 2.475 trillion CNY (0.35 trillion USD). The ratio of these amounts is 53 to 47%, highlighting a relatively high OOP payment burden for individuals (38, 39). In contrast to the practices in reference regions where drug price and expenses are indirectly managed (from P′ to P″ in Figure 1) and OOP caps are implemented, the absence of flexible access pathways (as shown in Figure 1) directly increases the financial burden on patients as drug prices and ACD expand. Consequently, even when high-value drugs are successfully included in the NRDL, patients may still face access barriers due to unaffordable OOP costs. This restricts their ability to choose costly innovative drugs. Under the decision-making framework in China’ BMI system, the amount of ACD represents the maximum annual cost level of a medication by its reasonable use. Reimbursing drugs (even if deemed cost-effective) with high ACD values without considering the financial capacity of the general population can lead to inequitable allocation of BMI funds, favoring wealthier patients and diseases associated with expensive treatments. To ensure the sustainable and efficient resources utilization, it is crucial to assess a drug’s affordability from the patient’s perspective. Thus, determining the maximum annual cost that average-income households can bear under BMI coverage can inform decisions on whether a drug is *too expensive*.

The ACD threshold indicates the point at which most households would encounter catastrophic financial risk, potentially causing patients to forgo innovative medication. Logistic regression analysis revealed that the marginal effect of ACD on catastrophic financial barriers likelihood peaked at 400,000 CNY (57,143USD), which is about 4.7 times the GDP per capita of China in 2022. This figure is much lower than the level of up to 12 times the local GDP per capita in the reference regions. The probability of experiencing catastrophic financial barriers exceeded 50% when ACD ranged from 440,000 to 450,000 CNY (62,857 to 64,286USD), approximately 5.2 times the GDP per capita of China in 2022, indicating the upper limit of affordability for average households. The cities included in the analysis, along with their respective insurance benefits, were highly representative, allowing us to identify a pragmatic threshold. By applying binary logistic regression innovatively, we introduced a quantitative reference for decision-making, enhancing equity and efficiency in informing reimbursement decisions from the perspective of patient affordability.

In the study, instead of using the catastrophic expenditure indicator, which defines catastrophic payments as exceeding 10% (or 25% as an alternative) of household consumption (40), we opted for a standard based on disposable income. While consumption expenditure may more accurately reflect the impact on living standards, we believe that, given the unique consumption and saving habits in China, as well as the tradition of family mutual assistance, using disposable income as the benchmark more accurately reflects the realities of the country. To accurately reflect the economic level and payment capacity in China, the disposable income of each province rather than that of the provincial capital cities were used. While in the context of insurance benefits, the UEBMI reimbursement rules from provincial capital cities and municipalities were chosen. The reimbursement rules adopted not only represent the current highest standards but also anticipate future enhancements within the BMI framework, reflecting future developments and the evolution of insurance benefits coordinated across BMI programs and geographical areas (41). Therefore, the selection of this data is well-justified, ensuring that the findings are sustainable and pertinent to China’s healthcare insurance system.

It is essential to acknowledge the existence of both the horizontal and vertical disparities in insurance benefits between the UEBMI and the URRBMI in China, along with the variations across provinces and municipalities. Furthermore, healthcare expenses extend beyond pharmaceutical costs to include, for instance, spending on medical services, diagnostic tests, and potential combination therapies. Therefore, the financial impact on patients during treatment could be underestimated, while the ACD threshold was overestimated. To address this, we performed subgroup analysis in economically disadvantaged regions to inform a more nuanced reference range for ACD constraints. By basing threshold settings on low-economic regions, financial risks can be mitigated and inter-regional fairness enhanced. Conversely, using national-level thresholds enables higher price ceilings for high-value drugs, increasing the likelihood of successful inclusion and improving patient access. We calculated the ACD value corresponding to a 50% probability of outcome events, providing decision-makers with a upper bound alert. This ACD value indicates that, under the current basic insurance reimbursement benefit levels, only high-income households may afford treatment with this specific drug and receive reimbursement, whereas average-income families may be priced out, leading to fairness concerns in fund allocation.

In addition, the findings of our study do not aim to capture the financial burden of healthcare on Chinese households, nor are they intended to predict catastrophic expenditure for individual households. By examining the relationships between annual costs, insurance benefits, and financial capacity, we established an evidence-based ACD reference to inform drug reimbursement decisions in China, complementing the economic appraisal formed by CUA, BIA and reference pricing. The findings offer a benchmark that enables payers to ascertain whether a drug is *too expensive*, thus necessitating the question of the opportunity cost of this medication within a constrained budget.

Nevertheless, the ACD threshold estimated in this study reveals that the entry prices of innovative pharmaceuticals in China’s BMI cannot be reconciled with the price levels reimbursed in other healthcare insurance systems. In the future, it is essential to establish a multi-stakeholder risk-sharing mechanism to balance the competing interests of innovative drug manufacturers’ profit expectations and patients’ access to these medications in China. For instance, Health insurance schemes can enhance financial sustainability by transitioning from a single-source to a multi-source funding system. Additionally, exploring outcome-based payment models can optimize financial efficiency for both insurance funds and patients. Furthermore, improving reimbursement benefits and establishing mechanisms, such as OOP caps or supplementary insurance products, to alleviate the financial burden of OOP expenses should be considered. Future research could focus on regional investigation and potentially analyze ACD thresholds at a provincial level, thereby providing a reference for the management of local reimbursement benefit scheme. To conclude, this innovative approach to estimating the threshold and integrating ACD evaluation into appraisals could provide important insights for reimbursement decision-making in other basic medical insurance systems worldwide, particularly those lacking OOP caps or safety net mechanism.

## Data Availability

The original contributions presented in the study are included in the article/Supplementary material, further inquiries can be directed to the corresponding authors.

## Footnotes

^1^Countries and regions were selected based on two criteria: (1) use of HTA frameworks for reimbursement decisions, and (2) inclusion in the international reference prices required by the National Healthcare Security Administration (NHSA) for NRDL adjustments. The regions include the UK, Germany, France, Canada, Australia, Sweden, Netherlands, Japan, the US, and South Korea. Sources included academic literature and documents obtained from the official websites of national healthcare organizations of each region.

^2^The exchange rate used in this study is 1 USD = 7 CNY.

^3^In reference regions, maximum costs accepted for high-value drugs (as GDP multiples) substantially exceed China’s, justifying an ACD upper limit of 1,200,000 CNY based on international benchmarks. With sample size contingent on ACD values assessed, a 50,000 CNY step size ensures a manageable sample size.

^4^Reimbursement rules and specific calculation methods are based on the National Healthcare Security Administration’s regulations; for detailed interpretation, refer to https://www.nhsa.gov.cn/art/2020/2/12/art_14_2571.html.
