# Supplementary material for: Should annual cost of the drug inform reimbursement decisions? A perspective from China’s healthcare security system
Source: Front Public Health. 2025 Apr 4;13:1552798. doi: 10.3389/fpubh.2025.1552798 (PMC12006103; doi:10.3389/fpubh.2025.1552798)
Supplement: Supplementary file 1 [file Table_1.docx]

**Supplementary Table 1-1.** Variable definitions

| Variables | Definition |
| --- | --- |
| **Outcome variables** | |
| the occurrence of catastrophic expenditure | Dummy variable: Whether a household experienced the defined catastrophic expenditure due to the ACD under the local reimbursement benefit; Yes = 1; No = 0 |
| **Independent variables** | |
| ACD | Continuous variable: the annual treatment cost of medication as specified in the drug's instructions; the value was set from the minimum of 50,000 RMB to a maximum of 1,200,000 RMB, with increments of 50,000 RMB between each value point, resulting in a total of 23 ACD values |
| **Control variables** |  |
| UEBMI reimbursement cap | Continuous variable: 31 values for municipalities and capital cities of each provinces |
| MSLME reimbursement cap | Continuous variable: 31 values for municipalities and capital cities of each provinces |
| UEBMI reimbursement rate | Categorical variable: the highest level = 3; the moderate level =2; the lowest level = 1 |
| MSLME reimbursement rate | Categorical variable: the highest level = 3; the moderate level =2; the lowest level = 1 |
| Co-payment ratio for Category B drug | Categorical variable: the portion of costs that patients must pay before reimbursement. If the rate is higher than 10%, level=0, Otherwise, level = 1 |

Note: All data for control variables were obtained from the municipal human resource and social security service platform and provincial or municipal government websites^[[1]](#footnote-0)^. The final update was completed in June 2024.

**Supplementary Table 1-2.** Sample Illustration of Observations

| Observation | Sample#1 | Sample#2 | …… | Sample#23 | Sample#24 | Sample#25 | …… | Sample#46 | …… | Sample#713 |
| --- | --- | --- | --- | --- | --- | --- | --- | --- | --- | --- |
| CITY | CITY#1 | CITY#1 | …… | CITY#1 | CITY#2 | CITY#2 | …… | CITY#2 | …… | CITY#31 |
| ACD | 50,000CNY | 10,000CNY | …… | 115,000CNY | 50,000CNY | 10,000CNY | …… | 115,000CNY | …… | 115,000CNY |
| UEBMI reimbursement cap | UEBMI reimbursement cap #1 | UEBMI reimbursement cap #1 | …… | UEBMI reimbursement cap #1 | UEBMI reimbursement cap #2 | UEBMI reimbursement cap #2 | …… | UEBMI reimbursement cap #2 | …… | UEBMI reimbursement cap #31 |
| MSLME reimbursement cap | MSLME reimbursement cap #1 | MSLME reimbursement cap #1 | …… | MSLME reimbursement cap #1 | MSLME reimbursement cap #2 | MSLME reimbursement cap #2 | …… | MSLME reimbursement cap #2 | …… | MSLME reimbursement cap #31 |
| UEBMI reimbursement rate | UEBMI reimbursement rate #1 | UEBMI reimbursement rate #1 | …… | UEBMI reimbursement rate #1 | UEBMI reimbursement rate #2 | UEBMI reimbursement rate #2 | …… | UEBMI reimbursement rate #2 | …… | UEBMI reimbursement rate 31 |
| MSLME reimbursement rate | MSLME reimbursement rate #1 | MSLME reimbursement rate #1 | …… | MSLME reimbursement rate #1 | MSLME reimbursement rate #2 | MSLME reimbursement rate #2 | …… | MSLME reimbursement rate #2 | …… | MSLME reimbursement rate #31 |
| Co-payment ratio for Category B drug | Co-payment ratio for Category B drug #1 | Co-payment ratio for Category B drug #1 | …… | Co-payment ratio for Category B drug #1 | Co-payment ratio for Category B drug #2 | Co-payment ratio for Category B drug #2 | …… | Co-payment ratio for Category B drug #2 | …… | Co-payment ratio for Category B drug #31 |
| OOP | OOP#1 | OOP#2 | …… | OOP#23 | OOP#24 | OOP#25 | …… | OOP#46 | …… | OOP#713 |

**Supplementary Table 1-3.** Example of OOP Expense Calculation Under City-Specific Reimbursement Rules

| Observation | Sample#i1:  ACDi1=50,000CNY | Sample#i4:  ACDi4=200,000CNY |
| --- | --- | --- |
| CITY#i | Rule#i | Rule#i |
| UEBMI deductible#i | 1,000CNY | 1,000CNY |
| MSLME deductible#i | 10,000CNY | 10,000CNY |
| UEBMI reimbursement cap #i | 50,000CNY | 50,000CNY |
| MSLME reimbursement cap #i | 200,000CNY | 200,000CNY |
| UEBMI reimbursement rate #i | 90% | 90% |
| MSLME reimbursement rate #i | 70% | 70% |
| Co-payment ratio for Category B drug #i | 10% | 10% |
| **The General Steps for OOP Calculation** | | |
| *Step1: for costs below the UEBMI reimbursement cap:* | 50,000CNY | 200,000CNY*(1-10%)-50,000CNY=130,000CNY |
| *Reimbursement payment 1=(ACD’*×*(1-co-payment ratio for Category B drugs)-deductible)*×*UEBMI reimbursement rate* | *Reimbursement payment 1=(*50,000CNY-1,000CNY)*90%=44,100CNY | *Reimbursement payment 1=(*50,000CNY-1,000CNY)*90%=44,100CNY |
| *Step2: for costs above the UEBMI reimbursement cap, below the MSLME reimbursement cap* | 0 | 130,000CNY+(50,000CNY-44,100CNY)=135,900CNY |
| *Reimbursement payment 2=((ACD’’-MSLME reimbursement cap)-MSLME deductible)*×*MSLME reimbursement rate* | 0 | *Reimbursement payment 2=*(135,900CNY-10,000CNY)*70%=88,130CNY |
| *Step3: Then, OOP=ACD-Reimbursement payment 1-Reimbursement payment 2* | OOPi1=50,000CNY-44,100CNY=5,900CNY | OOPi4=200,000CNY-44,100CNY-88,130CNY=67,770CNY |

Note: OOP calculation for each sample adheres to its specific reimbursement rules. All data for each reimbursement rule were obtained from the municipal human resource and social security service platform and provincial or municipal government websites.

1. The people’s republic of China government website: <https://www.gov.cn/;> the municipal human resource and social security service platform: [https://m12333.cn/.](https://m12333.cn/;) [↑](#footnote-ref-0)
