# Supplementary material for: Should annual cost of the drug inform reimbursement decisions? A perspective from China’s healthcare security system
Source: Front Public Health. 2025 Apr 4;13:1552798. doi: 10.3389/fpubh.2025.1552798 (PMC12006103; doi:10.3389/fpubh.2025.1552798)
Supplement: Supplementary file 2 [file Table_2.docx]

**Supplementary File 2.** **Subgroup analysis of twelve low-income provinces**

**Supplementary Table 2-1 Per capita disposable income of the twelve provinces**

| **Province** | **per capita disposable income in 2022 (CNY)** |
| --- | --- |
| Ningxia | 29599 |
| Shanxi | 29178 |
| Heilongjiang | 28346 |
| Henan | 28222 |
| Guangxi | 27981 |
| Jilin | 27975 |
| Xinjiang | 27063 |
| Qinghai | 27000 |
| Yunnan | 26937 |
| Xizang | 26675 |
| Guizhou | 25508 |
| Gansu | 23273 |

**Supplementary Table 2-2 The odds ratio results of the logistic regression analysis(N=276)**

| **Variable** | **Odds Ratio** | **SD** | **z** | **P** | **[95% Conf .Interval]** |
| --- | --- | --- | --- | --- | --- |
| ACD | 1.537732 | .1543731 | 4.29 | 0.000 | [1.263072, 1.872116] |
| UEBMI cap | .8357865 | .0432948 | -3.46 | 0.001 | [0.7550958, 0.9251] |
| UEBMI rate |  |  |  |  |  |
| 2 | .0096804 | 0154866 | -2.90 | 0.004 | [0.0004209, 0.2226571] |
| 3 | .0001247 | .0003059 | -3.67 | 0.000 | [1.02e-06, 0.0152658] |
| MSLME rate |  |  |  |  |  |
| 2 | .0017488 | .0038683 | -2.87 | 0.004 | [0.0000229, 0.1335262] |
| 3 | .0000116 | .0000384 | -3.45 | 0.001 | [1.81e-08, 0.0074606] |
| Constant | .2330526 | .4976387 | -0.68 | 0.495 | [0.0035471, 15.31194] |
| LR chi2 | | 302.52 | P | | 0.0000 |
| Log likelihood | | -18 .341499 | Pseudo R2 | | 0.8919 |

**Supplementary Table 2-3 The results of average marginal effects analysis(N=276)**

|  | **dy/dx** | **Std . Err .** | **z** | **P> \|z \|** | **[95% Conf .Interval]** |
| --- | --- | --- | --- | --- | --- |
| ACD | .0086867 | .0000244 | 356.24 | 0.000 | [0.0086389, 0.0087345 ] |
| UEBMI cap | -.0036212 | .0006191 | -5.85 | 0.000 | [-0.0048346, -0.0024078] |
| UEBMI rate |  |  |  |  |  |
| 2 | -.0936843 | .0237908 | -3.94 | 0.000 | [-0.1403134, -0.0470552] |
| 3 | -.1816208 | .0256398 | -7.08 | 0.000 | [-0.2318739, -0.1313676] |
| MSLME rate |  |  |  |  |  |
| 2 | -.1280606 | .0328173 | -3.90 | 0.000 | [-0.1923814, -0.0637398] |
| 3 | -.2293543 | .0393363 | -5.83 | 0.000 | [-0.306452, -0.1522567] |

**Supplementary Fig2-1. The curve of ACD average marginal effects in subgroup analysis**

**Supplementary Fig2-2. The curve of ACD cumulative effect in subgroup analysis**
